# Unhappiness Intensifies the Avoidance of Frequent Losses While Happiness Overcomes It

**DOI:** 10.3389/fpsyg.2016.01703

**Published:** 2016-11-02

**Authors:** Eldad Yechiam, Ariel Telpaz, Stas Krupenia, Anat Rafaeli

**Affiliations:** ^1^Faculty of Industrial Engineering and Management, Technion – Israel Institute of TechnologyHaifa, Israel; ^2^General Motors Research DepartmentHerzelia, Israel; ^3^Thales' Human Factors and Cognition LaboratoryDelft, Netherlands

**Keywords:** decisions from experience, happiness, rare events, individual differences, emotions

## Abstract

The implication of spontaneous and induced unhappiness to people's decision style is examined. It is postulated that unhappy individuals have a greater tendency to avoid frequent losses because these can have depleting effects, and unhappy individuals are more sensitive to such effects. This is evaluated in Study 1 by using an annoying customer call manipulation to induce negative affect; and by examining the effect of this manipulation on choices in an experiential decision task (the Iowa Gambling task). In Study 2 we examined the association between self-reported (un)happiness and choices on the same decision task. In Study 1 the induction of negative affect led to avoidance of choice alternatives with frequent losses, compared to those yielding rarer but larger losses. Specifically, this pertained to the advantageous alternatives with frequent vs. non-frequent losses. In Study 2 unhappiness was similarly associated with less exposure to frequent losses; while extreme high happiness was associated with no tendency to avoid frequent losses when these were part of an advantageous alternative. The findings clarify the role of happiness in decision making processes by indicating that unhappiness induces sensitivity to the frequency rather than to the total effect of negative events.

## Introduction

Positive-psychology theories of well-being and happiness often take an Aristotelian approach, in which happiness is the product of the pursuit for meaning in a person's life (Seligman and Csikszentmihalyi, 2000). In Aristotle's words “Happiness is the meaning and the purpose of life, the whole aim and end of human existence” (Aristotle, 2000). Consequently, these theories focus on variables such as moments of highly positive experiences at work (e.g., Csikszentmihalyi, 1990), people's relation to their work (Wrzesniewski and Dutton, 2001), the availability of time for personally significant activities (Perlow, 1999), and the fit between personal goals and one's personality (Kasser and Ahuvia, 2002). However, a much earlier Greek philosopher, Epicurus, had a different view on what makes people happy. He argued that well-being is simply the product of not-suffering (De Witt, 1964). In other words, happiness is the result of the absence of mental and physical pain. This concept resonates in modern Psychology as well, extending into Freud's pleasure principle (Freud, 1930) and being succinct in modern theories of motivation (e.g., Gray, 1994; Higgins, 1997). The current study assesses whether indeed people who are relatively unhappy have a different way of dealing with negative repercussions (i.e., penalties) than those in a happier state of mind. Specifically, we take a neo-epicurean approach that *happiness is associated with better capacity to emotionally withstand continuous negative repercussions*; and unhappiness leads to the reverse trend. Consequently, unhappy people tend to avoid small frequent penalties.

Previous findings have shown that people who are relatively unhappy have lower pain thresholds, and thus tend to avoid painful experiences to a greater extent. For instance, experimental studies demonstrated that a negative mood induced by imagery decreased pain tolerance while an elated mood increased pain tolerance (Hertel and Hekmat, 1994; Weisenberg et al., 1998; Tang et al., 2008). Similarly, people reporting more negative affectivity indicated more subjective distress as a result of pain experiences (Rhudy and Meagher, 2001; Zautra et al., 2005; Connelly et al., 2007). Finally, there is evidence of an association between the clinical diagnosis of major depression and the syndrome of chronic pain (Fishbain et al., 1986; Haythornwaite et al., 1991; Carroll et al., 2004). These findings suggest that unhappy individuals have lower capacity to withstand pain.

However, in studies of decision making the induction of negative affect using a variety of methods was found to increase the tendency to select risky alternatives producing gains and losses (e.g., Kuvaas and Kaufmann, 2004; Suhr and Tsanadis, 2006; Chuang and Lin, 2007). Similarly, adults and children with negative affectivity were typically found to exhibit more risk taking (e.g., Desrichard and Denarié, 2005; Garon and Moore, 2006; Sundqvist and Wennberg, 2015). Both sets of findings suggest that unhappy people are *less* sensitive to financial losses. A consistent finding emerges in studies focusing on the induction of positive affect, which demonstrated that *happy* people are *more* sensitive to financial losses (Isen and Patrick, 1983; Isen et al., 1988)^1^. Thus, one might conclude that relatively unhappy individuals show decreased, rather than increased, sensitivity to the psychological agitation inflicted by financial losses.

The current paper attempts to reconcile this apparent inconsistency by suggesting that the cognitive construct linked with unhappiness is not the sensitivity to the size of the loss, but rather the capacity to withstand frequent penalties. Pain tolerance studies examine the ability to withstand *continuous* pain in order to get a (formal or informal) reward at the end of the task. This experimental situation thus offers a tradeoff between frequent penalties as a result of the mounting pain and a one-time loss of one's (formal or informal) reward. We suggest that if decision tasks are similarly constructed, then unhappy individuals would avoid alternatives producing frequent losses.

Our predictions are consistent with previous findings showing that people with negative affect are more sensitive to depleting environmental consequences (Baumeister et al., 1998). The frequency of the negative event seems to be an important factor in the depletion process. For example, small frequent hassles were found to contribute more to people's stress and burnout than large life events (Lazarus et al., 1985; Zohar, 1997). Possibly, even minor financial losses have depleting effects when they are frequent. Indeed, in laboratory studies, people were found to weigh in the frequency of outcomes much more than their magnitude (Barron and Erev, 2003; Yechiam and Busemeyer, 2006; Camilleri and Newell, 2011). We posited that happy individuals would better tolerate the exposure to frequent losses; particularly if these lead to a net (accumulated) advantage in terms of one's outcomes, while unhappy individuals would tend to avoid frequent losses even in the latter circumstance because of their greater sensitivity to depleting factors.

Study 1 focused on experimentally induced negative affect, by means of an annoying customer call in a call-center simulation (c.f., Miron-Spektor and Rafaeli, 2009). Past research has shown that this protocol leads to a variety of negative emotions, particularly significant anger, and frustration (Miron-Spektor and Rafaeli, 2009), but also unpleasantness and stress (Gabay, 2008). It therefore appears that this manipulation is conducive to a state of negative well-being. In Study 2 we examined individual differences in unhappiness.

The dependent variable in both studies was the choice of alternatives yielding frequent as compared to rare losses. This was examined in the four-alternative Iowa Gambling task (Bechara et al., 1994), which manipulates in addition to the frequency of losses, the advantageousness of the choice alternative, allowing to examine the interaction between these factors.

## Study 1

Half of the participants heard a conversation between a customer service representative and an extremely dissatisfied and rude customer (Annoyance condition); the others heard a conversation that was identical in all other respects except that the customer expressed no particular emotion (Control condition). In the Annoyance condition the customer seemed hostile, nasty and critical; in the Control condition the customer was pragmatic with no clear sentiment. Additionally, following the annoying customer call participants reported their felt emotions (following Watson et al., 1988) as a manipulation check.

The main dependent variable was a novel index in the Iowa Gambling task (Bechara et al., 1994). The task involves repeated selections between four alternatives, whose outcomes are drawn from the fixed payoff distributions appearing in Table 1. We examined the choices from the two alternatives yielding frequent (50%) compared to infrequent (10%) losses. Across alternatives, the frequent loss alternatives (Loss50, DisLoss50) have the exact same expected value as their counterparts with less frequent losses (Loss10, DisLoss10). It was expected that individuals induced to be unhappy would demonstrate greater avoidance of the two frequent-loss options.

**Table 1:** **Outcome distributions for the choice alternatives of the Iowa Gambling task (Bechara et al., 1994)**.

**Alternative**	**Gains (tokens)**	**Losses (tokens)**	**EV**
Loss50	50 every time	50% chance of losing 50	25
Loss10	50 every time	10% chance of losing 250	25
DisLoss50	100 every time	50% chance of losing 250	−25
DisLoss10	100 every time	10% chance of losing 1250	−25

We also separately examined the frequent and less frequent loss options within each pair of advantageous alternatives (Loss50 vs. Loss10) and disadvantageous alternatives (DisLoss50 vs. DisLoss10). Again, within each pair the frequent and infrequent loss options have identical expected values. It was predicted that induced unhappiness would be associated particularly with the tendency to avoid the advantageous alternative with frequent minor losses compared to its counterpart with infrequent larger losses. In this case, exposure to frequent losses may be undertaken for the purpose of maintaining selections leading to net profit by individuals in the Control condition but less so by those induced to be unhappy. However, a contrasting prediction is that if one is selecting disadvantageously, then one values the disadvantageous alternatives more; and in this case within disadvantageous alternatives as well unhappiness should be associated with selection of the option with less frequent losses.

Additionally, for a subset of the participants we examined the degree of arousal during the task. Pupil diameter was used as a measure of arousal since it is considered an immediate and direct index of autonomic activation, which modulates cognitive and emotional processes (Andreassi, 2000; Bradley et al., 2008). We expected higher arousal in the Annoyance condition in line with the well-known relation between emotion and arousal (Routtenberg, 1968; Lindsley, 1970).

### Method

#### Participants

One-hundred and twenty undergraduate students (60 men and 60 women) participated in the study. We aimed for about 120 participants to provide sufficient power to detect small-to-medium sized effects. The participants' average age was 24 (ranging between 19 and 32). The data of two individuals was corrupted and they were removed from the analysis (leaving a total of 118). Participants were randomly assigned into the two experimental conditions, keeping an equal number of participants and gender proportions in each condition. Half of the participants performed the task with no physiological measures and half with the eye tracker (we refer to these two subsets as the Behavioral group and the Eye tracking group). The former received a basic fee of NIS 20 and additionally a performance-based stipend. The latter group received a basic fee of NIS 40 due to the longer procedure (which included calibration).

#### Study conditions

**Figure 1:**
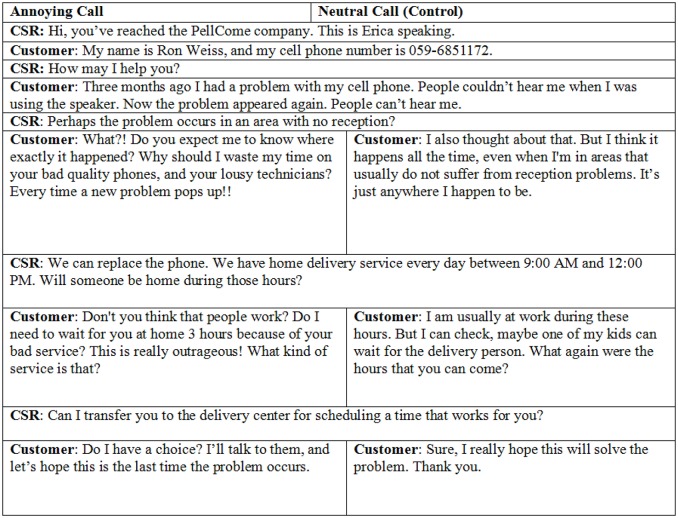
**Annoying vs. neutral conversations used in Study 1**.

The initial instructions that all participants received were as follows: “This experiment simulates a customer service center. As a Customer Service Representative you will be asked to perform several tasks, and will be compensated based on your performance. You will be asked to listen to a recorded conversation between a customer and another Customer Service Representative in your center, and then to freely recall it. Additionally, you will be asked to perform an unrelated decision-making task and respond to a short survey.” All participants listened to a pre-recorded conversation using headphones, which was different in the Annoyance and Control condition (see Figure 1).

The conversations in the calls were taken from transcripts of real customer service interactions in a local cellular provider (Miron-Spektor and Rafaeli, 2009). The problem reported by the customer was identical in both conditions, although the text was modified and included either negative expressions (Annoyance condition) or neutral ones (Control condition). Since anger is better recognized when expressed by males than females (Goos and Silverman, 2002), the customer in both conversations was a male and the service provider was a female.

### Measures

#### Iowa gambling task

A computerized version of the task devised by Bechara et al. (1994) was employed (see also Yechiam and Busemeyer, 2005). In this task, the participant sees four decks of cards, labeled A, B, C, and D, on a computer screen. Using the mouse, the participant repeatedly selects cards from any of the four decks. The amounts won and lost are displayed on the selected deck (see Table 1). The major dependent variable was the proportion of selection from the two alternatives yielding frequent losses (Loss50 + DisLoss50). As a secondary examination, we studied the difference between the proportion of selections from the alternatives with frequent or infrequent losses within the advantageous alternatives (Loss50 − Loss10) and disadvantageous (DisLoss50 − DisLoss10) alternatives. The original task instructions (Bechara et al., 1994) were used. Briefly, participants were not given prior information about the choice outcomes. To increase participants' motivation actual monetary amounts were used. Participants were told that every 100 points they win or lose would be converted to actual payments of 1 new Israeli Shekel. For the participants in the Behavioral group the task included 100 trials while for those in the Eye tracking group the task was shortened to 60 trials^2^.

#### Felt emotions

After hearing the conversation, participants were asked to indicate the extent to which to which they felt positive or negative emotions right now. A list of emotions was drawn from the PANAS scale (Watson et al., 1988). Following Harmon-Jones et al. (2009), the list included the three emotions: “angry,” “frustrated,” and “tranquil.” Additionally, for half the subjects we added the terms: “unpleasant,” “enthusiastic,” “attentive,” “stressed,” and “interested,” and for the remaining half we added the terms “energetic,” “exhausted,” “drowsy,” and “vibrant.” Questions were presented in a 5-point Likert scale (1 = very slightly or not at all to 5 = extremely). For the three affects that were administered to all subjects we created a single index of negative affect (reverse scoring the positive affect). The Cronbach's Alpha for this index was adequate (α = 0.72).

#### Pupil diameter

Pupil diameter was assessed as a measure of arousal. Eye-tracking data was collected using ViewPoint PC 60 EyeFrame system (Arrington Research, Scottsdale, Arizona). The system operates with a single tiny camera and an infrared illuminator aimed at the participant's dominant eye, and supported by comfortable head straps. It records pupil data at approximately 30 frames per second. Pupil data was measured as the diameter of the pupil during the customer-call. We also studied as a baseline measure the pupil diameter just after the calibration and prior to the start of the customer-call for a period of 30 s. During this time the participants were asked to look at the center of the screen and wait for the experimenter's instructions.

### Procedure

Those in the Eye tracking group first put on the eye tracker glasses, and the device was synchronized. From this point on the procedure was identical for both subgroups. Next, all participants listened to a pre-recorded conversation using headphones, which was the only difference between the Annoyance and Control condition (see Figure 1). They then reported their felt emotions, completed the Iowa Gambling task (Bechara et al., 1994), and performed a memory task that probed the contents of the conversation (for conciseness we do not report the results from this task). Participants were rewarded for correct recall of details in the memory task and for their selections on the Iowa Gambling task.

### Analysis

We conducted a manipulation check by examining the difference between conditions in felt emotions and pupil diameter. This was followed by an analysis of variance (ANOVA) examining the effect of condition (Annoyance and Control) and group (eye tracking vs. behavioral) on the rate of selection from alternatives with high-frequency losses in the Iowa Gambling task. Next, we separately examined the selection of advantageous and disadvantageous alternatives producing high-frequency losses. We then examined the effect of these factors on the rate of disadvantageous selections, for control purposes.

### Results and discussion

#### Felt emotions

Overall, participants reported more negative affect following the annoyance manipulation compared to the Control condition [*t*_(116)_ = 3.53, *p* <0.001]. In particular, in the Annoyance condition participants reported more anger [*t*_(116)_ = 3.78, *p* <0.001] and more frustration [*t*_(116)_ = 3.10, *p* = 0.002], as well as greater unpleasantness [*t*_(58)_ = 3.06, *p* = 0.002], and more stress [*t*_(58)_ = 2.10, *p* = 0.04]. Interestingly, lower and non-significant effects were registered for the positive emotions (e.g., calmness; *p* = 0.12; enthusiasm: *p* = 0.82).

#### Pupil diameter

Prior to the start of the customer-call there was no significant difference in pupil diameter between the Annoyance and Control condition [*t*_(56)_ = 1.53, *p* = 0.16]. However, during the conversation pupil diameter in the Annoyance condition was significantly larger [*t*_(56)_ = 3.31, *p* = 0.001], denoting an increase in arousal as a result of hearing its narrative.

#### Decision task

**Figure 2:**
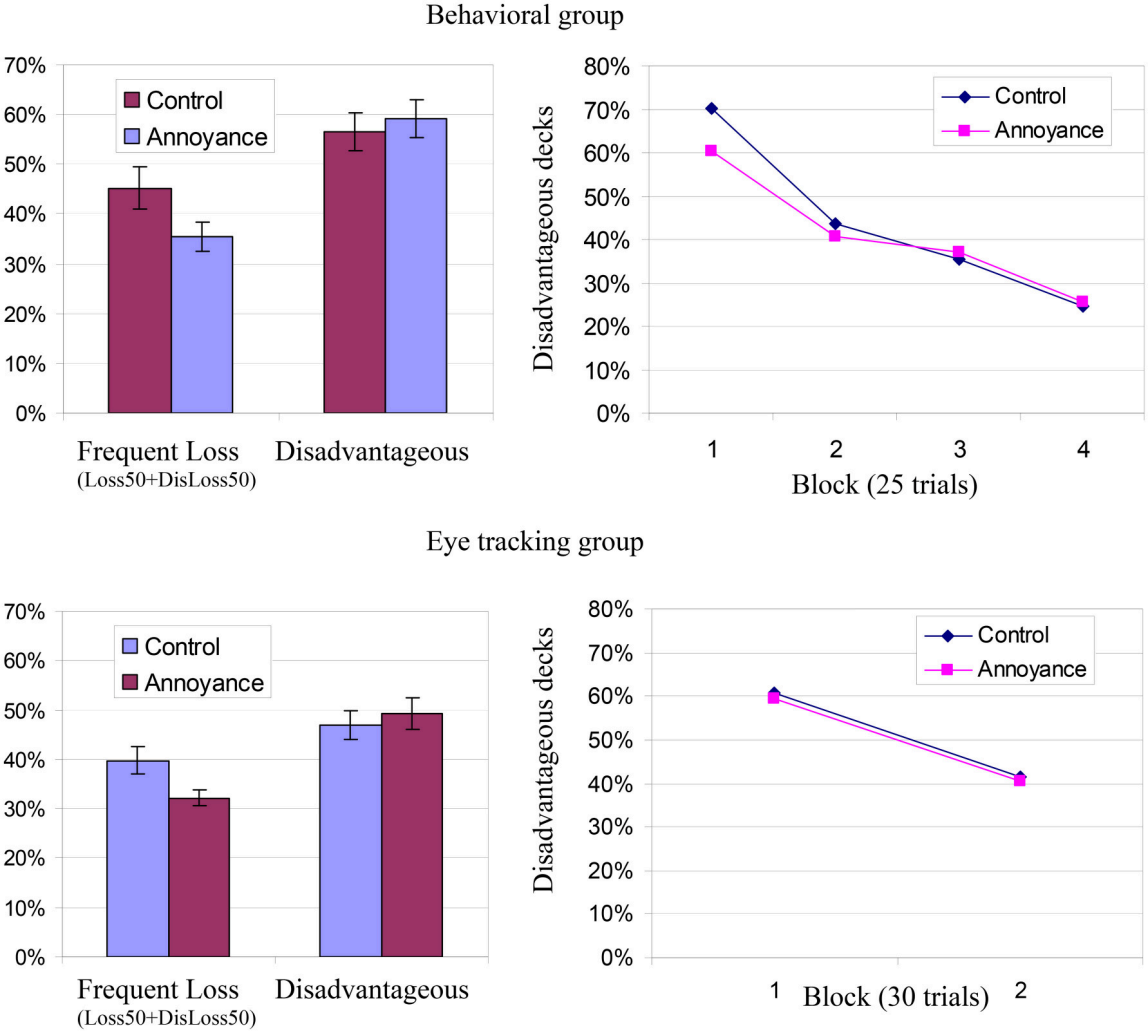
**Study 1 results. Top:** Results for participants in the Behavioral group. **Bottom**: Results for participants in the Eye tracking group. **Left**: Rate of selection from the two disadvantageous alternatives (DisLoss10 + DisLoss50) and from the two alternatives with frequent losses (Loss50 + DisLoss50) in the two experimental conditions (Annoyance and Control) across trials. Error terms denote confidence intervals. **Right**: Choices from the disadvantageous alternatives in four blocks of trials in the two experimental conditions.

Participants' learning curves on the Iowa Gambling task appear in Figure 2. As can be seen, on average they show the typical pattern of learning to select advantageously with repeated choices (as in Bechara et al., 1994). The proportions of selections from each alternative appear in Table 2. Across conditions and groups, alternatives with high-frequency losses were chosen less than those with infrequent losses. Alternative Loss50 was selected 21.8% of the time, compared to 31.4% selections of its advantageous counterpart, Loss10 [*t*_(129)_ = 3.73, *p* <0.001]. Alternative DisLoss50 was selected only 16.3% of the time, compared to 30.5% for DisLoss10 [*t*_(129)_ = 8.12, *p* <0.001]. We next examined the difference in choice behavior between the Annoyance and Control condition.

**Table 2:** **Choices in Study 1 for the participants in the Behavioral and Eye tracking groups: Averages and standard errors of selections from each alternative, in percents**.

	**Loss50**	**Loss10**	**DisLoss50**	**DisLoss10**
**BEHAVIORAL GROUP**				
Annoyance	21.0 ± 3.1	38.1 ± 3.8	14.4 ± 1.4	26.5 ± 3.0
Control	27.7 ± 4.0	28.8 ± 3.8	17.5 ± 3.2	25.9 ± 2.8
**EYE TRACKING GROUP**				
Annoyance	16.9 ± 1.0	32.3 ± 1.4	14.9 ± 0.5	35.0 ± 1.5
Control	21.3 ± 1.7	25.8 ± 1.2	18.5 ± 0.8	34.4 ± 1.7

The results (presented in Table 2 and left panes of Figure 2) showed a difference in the predicted direction. In both groups, participants in the Annoyance condition made fewer choices from the two frequent-loss alternatives (Loss50 + DisLoss50: 33.8% compared to 42.5%). An ANOVA showed that the effect of condition was significant [*F*_(1, 114)_ = 8.21, *p* = 0.005] while the effect of group (Behavioral vs. Eye tracking) and the interaction of group and condition were not significant, *F*_(1, 114)_ = 2.07, *p* = 0.15; *F*_(1, 114)_ = 0.13, *p* = 0.72. Thus, the negative affect induction resulted in less exposure to frequent losses.

We then proceeded into examining the advantageous and disadvantageous alternatives separately. The difference between conditions in relative selection from each of the two advantageous alternatives (Loss50 − Loss10) was significant in an ANOVA, *F*_(1, 114)_ = 7.01, *p* = 0.009, denoting a decreased tendency to select the frequent-loss alternative Loss50 compared to its advantageous counter-part Loss10 (which produces less frequent losses) for participants in the Annoyance condition. The effect of group (Behavioral vs. Eye tracking) and the interaction between group and condition, were not significant, *F*_(1, 114)_ = 0.03, *p* = 0.86; *F*_(1, 114)_ = 0.25, *p* = 0.62.

Such differences were not found for relative selection from the high and low- frequency disadvantageous alternatives, DisLoss50 − DisLoss10: [*F*_(1, 114)_ = 1.40, *p* = 0.24] and also not for the two disadvantageous alternatives together, DisLoss50 + DisLoss10: [*F*_(1, 114)_ = 0.48, *p* = 0.49]; suggesting that the manipulation specifically affected the exposure to frequent losses when they were advantageous.

We next examined whether participants' emotions can predict their avoidance of frequent losses. In this analysis we focused on the index for avoiding rare losses in advantageous selections (Loss50 − Loss10). As previously, the analysis was conducted separately for the two groups (Behavioral, Eye tracking). For the Behavioral group negative affect was negatively correlated with the Loss50 − Loss10 index (*r* = −0.26, *p* = 0.05)^3^, but such was not the case for the Eye tracking group (*r* = 0.19, *p* = 0.15) and across groups the effect was not significant. Given that participants only evaluated their emotions following the affective manipulation, individual differences in reported emotions represent a mixture of tonic and induction-specific effects, the interaction of which may have confounded the association with decision behavior. We therefore proceeded to examine the association between unhappiness and sensitivity to frequent losses with no affect induction.

## Study 2

In our next study we examined the association between self-reported (un)happiness and behavioral choices on the Iowa Gambling task. Our prediction was that negative well-being would be associated with a lower tendency to be exposed to frequent losses, particularly in advantageous alternatives (yielding greater avoidance of alternative Loss50 relative to Loss10).

### Method

#### Participants

One hundred and thirty undergraduate students (65 men and 65 women) participated in the study. We aimed for about 120 participants to provide sufficient power to detect small-to-medium sized effects and used all those showing up without excluding any participant. The participants' average age was 23.5 (ranging between 18 and 28). Their basic fee was NIS 20 and a performance-based stipend.

### Procedure

We administered the Iowa Gambling task (Bechara et al., 1994) using the same instructions as in the previous study. Following the task participants completed the Oxford Happiness Questionnaire—Shortened version (Hills and Argyle, 2002). This questionnaire has 10 items with sample questions being “I feel that life is very rewarding” and “I don't feel particularly pleased with the way I am” (reversed-scored)^4^. It is answered on a six-point Likert scale ranging from “agree strongly” (1) to “disagree strongly” (6). Three items are reversed scored. Scores range from 8 to 48, with higher scores on the scale indicating a greater level of happiness. As the responses were negatively skewed, and given the sensitivity of Cronbach's Alpha to skewed results (Dunlap et al., 1994), we examined reliability using log transformed scores. The inter-item reliability was adequate (α = 0.84).

### Analysis

Our main analysis examined the association between self-reported happiness on the Oxford Happiness Questionnaire and the rate of selection from the alternatives with high-frequency losses in the Iowa Gambling task. Again, this was followed by the separate examination of disadvantageous and disadvantageous alternatives with high-frequency losses. For comparison, we also examined the association with the rate of disadvantageous selections. All analyses were conducted using Pearson correlation but were replicated using Spearman's rank order correlation.

### Results and discussion

As previously, alternatives with high-frequency losses were chosen less than those with infrequent losses. Alternative Loss50 was selected 26.3% of the time, compared to 34.4% selections of Loss10 [*t*_(129)_ = 2.52, *p* = 0.01]. Alternative DisLoss50 was selected only 12.5% of the time, compared to 26.3% for DisLoss10 [*t*_(129)_ = 10.91, *p* <0.001].

**Figure 3:**
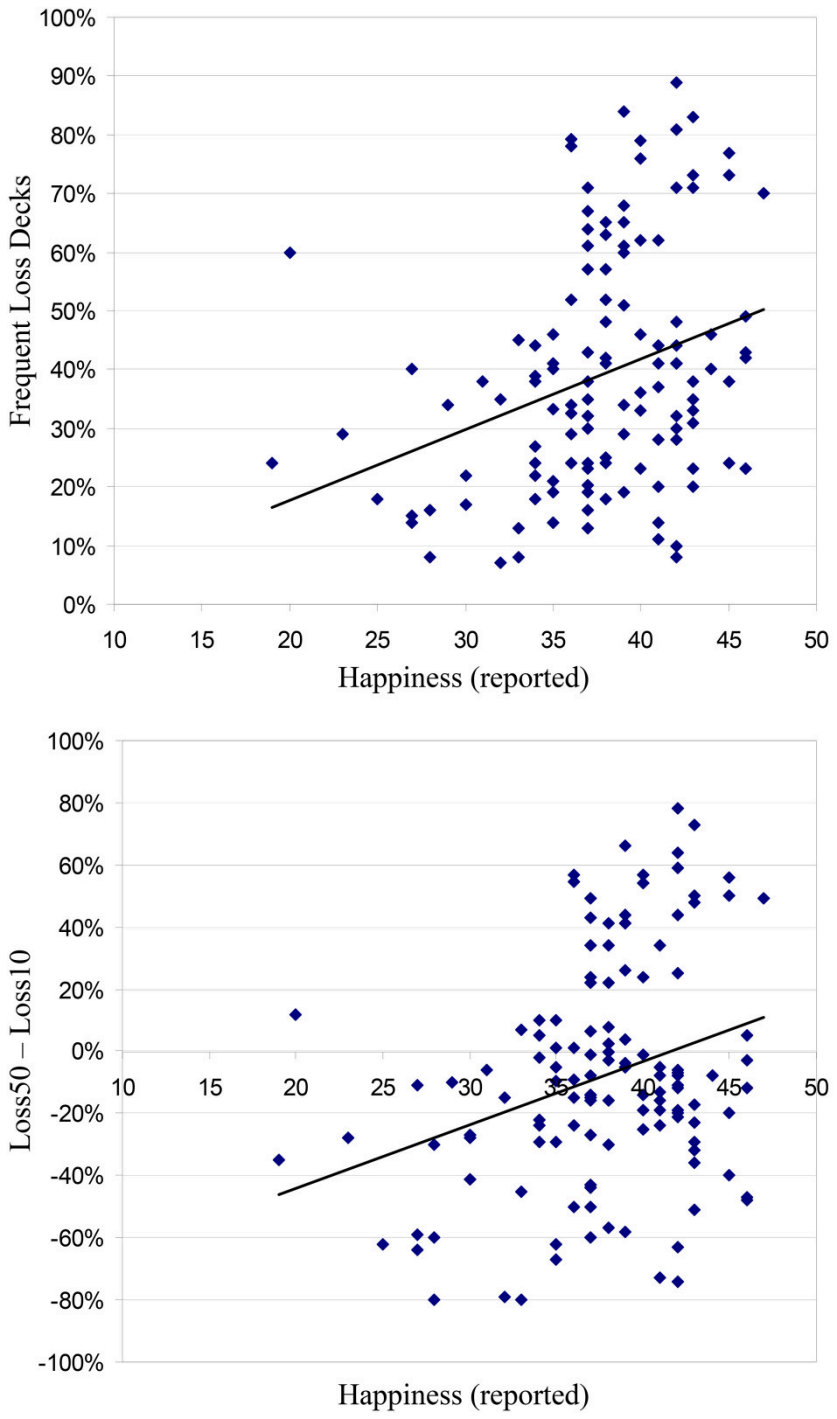
**Study 2 results**. A scatter point of the relation between self-reported well-being on the Oxford Happiness Scale (Hills and Argyle, 2002) and choice behavior. **Top**: Association with rate of selections from the two alternatives with frequent losses (Loss50 + DisLoss50). **Bottom**: Association with relative choices from alternatives Loss50 and Loss10 (Loss50 − Loss10). The trend-lines represent the results of a linear regression between the two variables.

Figure 3 top panel shows a scatter plot of the relation between self-reported happiness and the rate of selection from the two alternatives with high frequency negative outcomes (Loss50 + DisLoss50). The correlation between these two indices was positive and significant, *r* = 0.31, *p* <0.001, marking a tendency of low happiness individuals to avoid high frequency losses. Additionally, as shown in Figure 3 bottom panel, the association between happiness ratings and the Loss50 − Loss10 index was significant, *r* = 0.30, *p* <0.001. Both correlations were replicated using Spearman's rho: Happiness with Loss50 + DisLoss50, *r* = 0.34, *p* <0.001; and with Loss50 − Loss10, *r* = 0.25, *p* = 0.004. Thus, relatively unhappy individuals specifically tended to avoid the advantageous option producing high-frequency losses, and preferred the alternative with lower frequency losses. By contrast, happiness ratings were not associated with relative selection from the disadvantageous alternative producing high-frequency losses compared to its counter-part (DisLoss50 − DisLoss10), *r* = 0.13, *p* = 0.13. The results also revealed no significant correlation between happiness ratings and the combined score of disadvantageous selections (DisLoss50 + DisLoss10), *r* = 0.08, *p* = 0.39.

The current results are consistent with our prediction that unhappy individuals tend to avoid high-frequency losses, while happy individuals allow themselves to be exposed to these losses when this is advantageous. Indeed, as indicated in Figure 3 bottom panel, from a certain level of happiness (a score of 42 and above on the Oxford Happiness Questionnaire; representing about 28% of the sample) individuals no longer avoided the frequent-loss advantageous alternative compared to its counter-part with infrequent losses.

## Discussion

In Study 1 the communicated emotional expressions of another person were used as a powerful inducer of negative affect (following Kopelman et al., 2006). The results of this study showed that an annoying customer call in a customer-service simulation (Miron-Spektor and Rafaeli, 2009) increased the avoidance of alternatives producing minor but frequent losses. Thus, the annoyance manipulation led to increased sensitivity to the frequency and not to the size of the losses. In Study 2 we find similar results for individual differences: Individuals who self-rated as relatively unhappy avoided alternatives producing minor but frequent losses and opted for ones producing larger but rarer losses. This finding suggests that the relation between happiness and the exposure to frequent losses occurs irrespectively of a particular affect induction technique.

In both studies, the effect was stronger for advantageous alternatives, and it was not statistically significant within disadvantageous alternatives. One possible reason for this interaction is that happier individuals were willing to tolerate frequent losses if they led to positive net outcomes over repeated selections, but not otherwise. By contrast, for unhappier individuals this willingness to tolerate frequent losses for a purpose was compromised.

Our results do leave some ambiguity with regards to the specific affect associated with avoidance of frequent losses. Our correlational analysis in Study 1 indicated a significant association between negative affect and the avoidance of frequent losses in one of the studied groups but not in the other, while examination of specific items showed that different negative emotions led to similar associations with the avoidance of frequent losses, thus suggesting that further research should be conducted to identify the specific emotions modulating the avoidance of rare losses. For instance, though our manipulation led to several negative emotions, we did not study the effect of fear emotions which seems to arouse a unique pattern of risk aversion (Lerner and Keltner, 2001). Furthermore, the specific processes leading to the sensitivity to frequent losses could be investigated further. For instance, one could differentiate the sensitivity to the frequency of losses *per se* or from a recency effect whereby one is more affected by the most recent outcomes (which in the case of an alternative with frequent losses tend to be negative). Still, it does not seem likely that the current result are driven by a mere recency effect (independently of gaining and losing) because this would imply an association between (un)happiness and disadvantageous selection in the Iowa Gambling task (see Yechiam and Busemeyer, 2005); such an association was not found in the present study.

The current results shed light on the apparent paradox that unhappy individuals have lower pain tolerance (e.g., Hertel and Hekmat, 1994; Weisenberg et al., 1998; Tang et al., 2008) while exhibiting more risk taking in laboratory tasks involving losses. The former finding can be interpreted as higher sensitivity to negative stimuli on the part of unhappy individuals, while the latter can be interpreted as lower sensitivity to them. We have shown that when a decision task includes an alternative with *frequent losses*, unhappy individuals are less willing to be exposed to these losses and prefer instead to receive less frequent but larger losses.

Another contribution of our study is in finding a boundary condition for the increased weighting of frequent compared to rare events (also known as the underweighting of rare events; Barron and Erev, 2003; Rakow and Newell, 2010; Di Guida et al., 2015). In both studies, the tendency to avoid advantageous frequent losses was curbed by (relative) positive affect and well-being. Most striking was the finding that high-happiness individuals no longer opted for rare large penalties over frequent penalties, thus showing no underweighting of rare events. We therefore suggest that affective depletion may be one of the mechanisms leading to preference of rare over common penalties. By affective depletion we refer to emotional exhaustion which typically results from repeated negative experiences (Wright and Cropanzano, 1998)^5^. The notion that affective depletion contributes to the underweighting of rare events is consistent with the findings that underweighting of rare events is smaller in the gain domain where there are no depleting losses (e.g., Barron and Erev, 2003).

A common modern view concerning emotions is that they can both improve and impair decision making (e.g., Shiv et al., 2005; Baumeister et al., 2007; Loewenstein, 2010). For example, research has shown that individuals with lesioned ventromedial frontal cortices that have difficulties associating their emotions with anticipated consequences of their actions, display excessive risk taking and perform poorly in the Iowa Gambling task (Bechara et al., 1994). Yet subsequent research has shown that if the task is changed so that the risky alternatives are advantageous the same frontal lesioned individuals perform better than healthy controls (Shiv et al., 2005). Nevertheless, even proponents of the argument that emotions can both improve and impair decision making often suggest a deleterious role for current emotions, as opposed to anticipated emotions marking one's emotional perception (Baumeister et al., 2007). By contrast, in the present study the avoidance of frequent losses was independent of people's risk taking or advantageous selection. For example, in all study conditions our findings show the typical pattern of moving from disadvantageous to advantageous selection in the Iowa Gambling task. Thus, we find that current emotions did not have a negative effect on task performance. This suggests that as for anticipated emotional consequences, current emotions can predispose individuals to select certain types of incentives, but the advantageousness of these incentive structures seems to depend on the relevant task characteristics.

Additionally, current emotions may have counteracting effects on the ability to pay attention and allocate cognitive resources. On the one hand, as suggested by Baumeister et al. (2007) negative emotions toward an external object (e.g., anger) may take away the focus from tasks conducted following this emotion. Attention may wander to the prior negative experience, or to self-related thoughts (Kanfer and Ackerman, 1989), which may reduce on-task attention, and consequently impair cognitive scrutiny and performance. On the other hand, negative affective reactions may also increase the overall attentional pool (Taylor, 1991) and this can lead to improved performance in subsequent tasks. For instance, financial losses experienced during a decision task were found to lead to more advantageous decisions in an unrelated task performed in subsequent trials (Yechiam and Hochman, 2013). Thus, the relation between negative emotions and cognitive effort may be more complicated than previously suggested, and could depend on moderators such as one's current pool of attention, and the degree to which one treats subsequent experience following a negative affect as part of a general context warranting attention^6^. The examination of such moderators is an interesting question for subsequent research.

## Conclusions

The current findings show that performance in a basic task of choosing between reinforcements is affected by one's happiness. As opposed to previous studies, we highlighted the role of the frequency of negative reinforcements. In our two studies relatively unhappy individuals preferred to accept high magnitude losses compared to high frequency losses of the same overall expected value. These findings suggest a psychological moderator to the well-known phenomenon whereby people are sensitive to recurring losses and prefer receiving larger to more frequent ones. This tendency is aggravated under conditions of duress, but is reduced for individuals reporting high positive well-being.

## Author contributions

All authors contributed to the design of the studies and the concept of the paper. AT and SK coordinated the studies. EY conducted the analyses and wrote and initial draft of the paper to which all authors contributed.

### Conflict of interest statement

The authors declare that the research was conducted in the absence of any commercial or financial relationships that could be construed as a potential conflict of interest.

## Footnotes

^1^This pattern was obtained with descriptive lotteries, and hence it is usually explained by the idea that people who are feeling relatively happy are more motivated to maintain their current state and thus may have more to lose by taking risk than unhappier individuals (e.g., Isen and Simmonds, 1978).

^2^This was due to a technical difference between the versions used in two labs.

^3^From the three affects indexed, the highest correlation was with anger, *r* = −0.27, *p* = 0.04; the lowest with frustration, *r* = −0.20, *p* = 0.14.

^4^The questionnaire was administered after the Iowa Gambling task. Thus, across studies the design is counter-balanced for the order of the task and the reporting of emotional state.

^5^Most typically, affective depletion has been studied in job settings where frequency is defined in resolution of days (e.g., Lazarus et al., 1985; Zohar, 1997; Wheeler et al., 2013) with findings showing that it is associated with stress, burnout, and somatic symptoms. However, we suggest that emotionally depleting effects of repeated negative experiences also emerge in a small scale when negative outcomes are presented over and over again within a short time.

^6^For example, if a customer service operator in a call-center received an insult from a customer, then her attention to the next customer call could be very different if she interpreted the insult as an ongoing problem which may be relevant to the forthcoming call, or as a single contained incident. In the former case she may be driven to pay more attention than usual to the next call whereas in the latter she may mind wander about the prior negative event, which could take her focus away from the upcoming call.
